# Supplementary material for: Pre-epidemic evolution of the MRSA USA300 clade and a molecular key for classification
Source: Front Cell Infect Microbiol. 2023 Jan 24;13:1081070. doi: 10.3389/fcimb.2023.1081070 (PMC9902376; doi:10.3389/fcimb.2023.1081070)
Supplement: Supplementary file 5 [file DataSheet_1.pdf]

Supplemental: 6 tables and 8 figures

Table S1: Genome quality statistics for newly reported genomes generated using QUAST.

	**Assembly**			
**Statistic**	**2m-n**	**BK2448**	**BK2561B**	**65_669**
# contigs (>= 0 bp)	75	64	77	26
# contigs (>= 1000 bp)	38	28	34	19
# contigs (>= 5000 bp)	32	24	26	12
# contigs (>= 10000 bp)	25	18	23	11
# contigs (>= 25000 bp)	19	15	20	10
# contigs (>= 50000 bp)	16	12	17	9
Total length (>= 0 bp)	2899456	2874324	2871754	2777101
Total length (>= 1000 bp)	2892994	2867581	2864850	2775911
Total length (>= 5000 bp)	2867348	2852203	2838817	2753096
Total length (>= 10000 bp)	2827327	2814495	2815656	2747552
Total length (>= 25000 bp)	2718877	2760373	2753311	2726838
Total length (>= 50000 bp)	2605280	2643903	2646126	2684464
# contigs	38	30	34	19
Largest contig	343261	610876	677524	883770
Total length	2892994	2869164	2864850	2775911
GC (%)	32.86	32.87	32.73	32.86
N50	198538	300945	202206	566023
N75	112194	147425	113145	348456
L50	5	4	4	2
L75	11	7	9	4
# N's per 100 kbp	20.81	19.87	7.54	4.29
All statistics are based on contigs of size >= 500 bp, unless otherwise noted (e.g., "# contigs (>= 0 bp)" and "Total length (>= 0 bp)" include all contigs).				

Table S2: General outline of methods for finding similar genomes using our bait genones (BK2651, V2200, M121, 65_669). Note, we set the number of genomes to be reported (100 for WhatsGNU and 50 for PATRIC).

	BK2651	V2200	M121	65_669
Reason:	**Earliest USA300**	**Well-characterized early USA300**	**Well-characterized SAE isolate**	**Earliest divergent NAE isolate**
Number of genomes from WhatsGNU: Similar genome finder based on protein sequence	100	100	100	100
Number of genomes from PATRIC: Similar genome finder based on nucleotide sequence	50	50	50	50
Total number of genomes found:	600			
Number of genomes after removing replicates:	204			
Number genomes deemed contamination free by MASH:	198			

Table S3: Ancestral state reconstruction was done as described in Fig. 3. To test for the impact of sampling bias we randomly chose 11 EB genomes from North America and reassigned their geographical location as “unknown”, resulting in an equal number of EB genomes (17 each) with North American and South American assignments. We repeated this procedure 10 times. This table shows the probability of location for each node in the EB, nodes are labeled in Fig. S4

run1								
node	Africa	Asia	Australia	Europe	North	South	Trinidad	Unknown
n22	0.0156	0.0052	0.0099	0.0949	0.4111	0.2047	0.0289	0.2297
n3	0.0153	0.0051	0.0102	0.0933	0.4075	0.2042	0.0282	0.2361
n33	0.0153	0.0051	0.0103	0.0933	0.4075	0.2045	0.0282	0.2357
n4	0.0153	0.0051	0.0106	0.0932	0.4073	0.2058	0.0281	0.2346
n8	0.0153	0.0051	0.0110	0.0934	0.4077	0.2078	0.0281	0.2316
n88	0.0152	0.0050	0.0125	0.0927	0.4067	0.2135	0.0277	0.2266
n99	0.0149	0.0049	0.0148	0.0915	0.4044	0.2222	0.0271	0.2203
n123	0.0155	0.0052	0.0098	0.0957	0.4101	0.2038	0.0307	0.2292
run2								
node	Africa	Asia	Australia	Europe	North	South	Trinidad	Unknown
n22	0.0154	0.0052	0.0098	0.0942	0.4230	0.2040	0.0289	0.2196
n3	0.0152	0.0051	0.0101	0.0927	0.4210	0.2035	0.0282	0.2243
n33	0.0152	0.0051	0.0102	0.0927	0.4209	0.2038	0.0282	0.2239
n4	0.0151	0.0051	0.0105	0.0926	0.4209	0.2050	0.0281	0.2227
n8	0.0151	0.0051	0.0109	0.0928	0.4210	0.2069	0.0281	0.2200
n88	0.0150	0.0050	0.0124	0.0921	0.4204	0.2124	0.0278	0.2149
n99	0.0148	0.0049	0.0147	0.0909	0.4167	0.2207	0.0272	0.2101
n123	0.0153	0.0051	0.0097	0.0950	0.4226	0.2031	0.0306	0.2186
run3								
node	Africa	Asia	Australia	Europe	North	South	Trinidad	Unknown
n22	0.0059	0.0020	0.0039	0.0541	0.3481	0.3789	0.0291	0.1780
n3	0.0016	0.0005	0.0011	0.0219	0.3709	0.4532	0.0043	0.1464
n33	0.0016	0.0005	0.0010	0.0213	0.3644	0.4534	0.0038	0.1539
n4	0.0016	0.0005	0.0011	0.0226	0.3248	0.4730	0.0029	0.1735
n8	0.0020	0.0007	0.0014	0.0287	0.3291	0.4737	0.0027	0.1617
n88	0.0023	0.0008	0.0016	0.0244	0.3445	0.4846	0.0022	0.1395
n99	0.0023	0.0008	0.0016	0.0212	0.3569	0.5131	0.0019	0.1022
n123	0.0056	0.0019	0.0037	0.0694	0.2904	0.3680	0.0734	0.1874
run4								
node	Africa	Asia	Australia	Europe	North	South	Trinidad	Unknown
n22	0.0153	0.0051	0.0098	0.0936	0.4140	0.2027	0.0284	0.2311
n3	0.0151	0.0050	0.0100	0.0922	0.4161	0.2023	0.0277	0.2316
n33	0.0151	0.0050	0.0101	0.0921	0.4161	0.2026	0.0277	0.2313
n4	0.0150	0.0050	0.0104	0.0920	0.4161	0.2038	0.0276	0.2300
n8	0.0150	0.0050	0.0109	0.0922	0.4147	0.2058	0.0276	0.2288
n88	0.0149	0.0050	0.0124	0.0915	0.4120	0.2115	0.0272	0.2255
n99	0.0147	0.0048	0.0146	0.0903	0.4081	0.2201	0.0266	0.2207
n123	0.0152	0.0051	0.0096	0.0944	0.4116	0.2018	0.0302	0.2321
run5								
node	Africa	Asia	Australia	Europe	North	South	Trinidad	Unknown
n22	0.0048	0.0016	0.0032	0.0411	0.4884	0.3277	0.0237	0.1096
n3	0.0010	0.0003	0.0007	0.0138	0.5371	0.3724	0.0026	0.0721
n33	0.0009	0.0003	0.0006	0.0133	0.5387	0.3707	0.0023	0.0731
n4	0.0009	0.0003	0.0006	0.0133	0.5470	0.3779	0.0015	0.0585
n8	0.0012	0.0004	0.0008	0.0183	0.5505	0.3744	0.0015	0.0530
n88	0.0013	0.0004	0.0009	0.0142	0.5698	0.3740	0.0012	0.0382
n99	0.0014	0.0005	0.0010	0.0129	0.5536	0.3967	0.0011	0.0328
n123	0.0047	0.0016	0.0031	0.0520	0.4265	0.3359	0.0614	0.1149
run6								
node	Africa	Asia	Australia	Europe	North	South	Trinidad	Unknown
n22	0.0154	0.0052	0.0098	0.0942	0.4154	0.2036	0.0287	0.2277
n3	0.0152	0.0051	0.0101	0.0927	0.4211	0.2032	0.0280	0.2247
n33	0.0152	0.0051	0.0102	0.0927	0.4207	0.2035	0.0280	0.2248
n4	0.0151	0.0051	0.0105	0.0926	0.4198	0.2047	0.0279	0.2244
n8	0.0151	0.0051	0.0110	0.0927	0.4176	0.2067	0.0278	0.2239
n88	0.0150	0.0050	0.0124	0.0920	0.4145	0.2123	0.0275	0.2212
n99	0.0148	0.0049	0.0147	0.0908	0.4098	0.2210	0.0269	0.2171
n123	0.0153	0.0051	0.0097	0.0950	0.4120	0.2027	0.0305	0.2296
run7								
node	Africa	Asia	Australia	Europe	North	South	Trinidad	Unknown
n22	0.0155	0.0052	0.0099	0.0945	0.4145	0.2040	0.0288	0.2277
n3	0.0152	0.0051	0.0102	0.0930	0.4137	0.2036	0.0281	0.2312
n33	0.0152	0.0051	0.0102	0.0929	0.4132	0.2039	0.0281	0.2313
n4	0.0152	0.0051	0.0106	0.0928	0.4120	0.2052	0.0280	0.2312
n8	0.0152	0.0051	0.0110	0.0930	0.4118	0.2071	0.0280	0.2289
n88	0.0151	0.0050	0.0125	0.0923	0.4100	0.2128	0.0276	0.2247
n99	0.0149	0.0049	0.0147	0.0911	0.4073	0.2214	0.0270	0.2187
n123	0.0154	0.0052	0.0097	0.0953	0.4145	0.2031	0.0306	0.2262
run8								
node	Africa	Asia	Australia	Europe	North	South	Trinidad	Unknown
n22	0.0155	0.0052	0.0098	0.0944	0.4233	0.2042	0.0290	0.2186
n3	0.0152	0.0051	0.0101	0.0929	0.4261	0.2037	0.0283	0.2186
n33	0.0152	0.0051	0.0102	0.0929	0.4260	0.2040	0.0283	0.2184
n4	0.0152	0.0051	0.0105	0.0927	0.4264	0.2052	0.0282	0.2167
n8	0.0152	0.0051	0.0110	0.0929	0.4245	0.2071	0.0282	0.2160
n88	0.0151	0.0050	0.0124	0.0923	0.4213	0.2126	0.0279	0.2135
n99	0.0148	0.0049	0.0147	0.0911	0.4170	0.2210	0.0273	0.2092
n123	0.0154	0.0052	0.0097	0.0952	0.4217	0.2034	0.0307	0.2188
run9								
node	Africa	Asia	Australia	Europe	North	South	Trinidad	Unknown
n22	0.0154	0.0052	0.0098	0.0942	0.4148	0.2036	0.0287	0.2284
n3	0.0152	0.0051	0.0101	0.0926	0.4152	0.2031	0.0280	0.2308
n33	0.0152	0.0051	0.0102	0.0926	0.4155	0.2034	0.0279	0.2301
n4	0.0151	0.0050	0.0105	0.0925	0.4163	0.2047	0.0278	0.2280
n8	0.0151	0.0050	0.0109	0.0927	0.4152	0.2067	0.0278	0.2265
n88	0.0150	0.0050	0.0124	0.0920	0.4128	0.2123	0.0275	0.2229
n99	0.0148	0.0049	0.0147	0.0908	0.4090	0.2209	0.0269	0.2180
n123	0.0153	0.0051	0.0097	0.0949	0.4129	0.2027	0.0304	0.2289
run10								
node	Africa	Asia	Australia	Europe	North	South	Trinidad	Unknown
n22	0.0052	0.0017	0.0035	0.0477	0.3650	0.4281	0.0245	0.1242
n3	0.0013	0.0004	0.0009	0.0179	0.3650	0.5109	0.0033	0.1004
n33	0.0012	0.0004	0.0008	0.0173	0.3669	0.5096	0.0029	0.1009
n4	0.0012	0.0004	0.0008	0.0175	0.3682	0.5207	0.0021	0.0891
n8	0.0016	0.0005	0.0010	0.0231	0.3655	0.5184	0.0020	0.0878
n88	0.0019	0.0006	0.0012	0.0198	0.3685	0.5231	0.0017	0.0832
n99	0.0019	0.0006	0.0013	0.0172	0.3703	0.5439	0.0015	0.0633
n123	0.0049	0.0016	0.0033	0.0616	0.3422	0.4120	0.0618	0.1126

Table S4: Characterization of *cap5D* and *cap5D* mutations

Allele	Mutation (nt)	Mutation Nature (AA/ protein)	Active Enzyme?
cap5E_223	G223T	Asp75Tyr in enzyme active site	No
cap5E_223_A170G	A170G, G223T	Tyr57Cys, Asp75Tyr in enzyme active site	No
cap5E_223_T17C	T17C, G223T	Lle6Thr, Asp75Tyr in enzyme active site	No
cap5E_223_G117A	G117A, G223T	silent, Asp75Tyr in enzyme active site	No
cap5E_WT	None	wt	Yes
cap5E_T116C	T166C	Leu58Phe	?
cap5E_223_G268C	G223T, G268C	Asp75Tyrin enzyme active site, Glu88Gln	No
cap5E_A85G	A85G	Lle28Val	?
cap5E_T458C	T458C	silent- wt	Yes
cap5E_G613A	G613A	Ala205Thr	?
cap5E_A137**Δ**	A137delete	Frameshift	No (?)
cap5E_518AG	518AG	Frameshift	No (?)
cap5D_994AA	994AA	Frameshift, truncated Cap5D	No
cap5D_994AA_C737T	C737T, 994AA	Frameshift, truncated Cap5D	No
cap5D_994AA_G415A	G415A, 994AA	Frameshift, truncated Cap5D	No
cap5D_994AA_G555A	G555A, 994AA	Frameshift, truncated Cap5D	No
capDE_WT	None	wt	Yes
cap5D_**Δ**210 nt	Missing 210nt	no protein made	No (?)
no *cap5D* found	NA	NA	No (?)

Table S5: Novel protein alleles specific to USA300-PEB1 clade. *mutation from MRCA with EB/SAE/NAE clades to PEB1

Gene	Protein	Mutation* (nt)	Mutation* Nature (AA/ protein)	Mutation Tolerated_SIFT	Mutation Tolerated_dezyme	PBD (WT)
hisB	Imidazoleglycerol-phosphate dehydratase	C158G	Ala53Gly	No	No	2AE8

Table S6: Novel protein alleles specific to USA300-SAE and NAE clades. *=mutation from MRCA with non-epidemic genomes.

Gene	Protein	Mutation* (nt)	Mutation* Nature (AA/ protein)	Mutation Tolerated_SIFT
der	GTPase Der	A1094G	D365G	Yes
desR	Transcriptional regulatory protein DesR	A154T	I52F	Yes
nagE	PTS system N-acetylglucosamine-specific EIICBA component	C224T	A75V	Yes
comEC	Natural transformation protein	G608T	G203V	No
sdcS	Sodium-dependent dicarboxylate transporter SdcS	C712T	H238Y	No

Table S7: Basis for deriving sensitivity and specificity. Table showing positive and negative values for A. USA300 or B. NAE. MK= molecular key

A

	USA300 on Tree	Not USA300 on Tree
**USA300 on MK**	**True Postive: 4,069**	**False Positive: 1**
**Not USA300 on MK**	**False Negative: 7**	**True Negative: 38,732**

B

	NAE on Tree	Not NAE on Tree
**NAE on MK**	**True Postive: 3,945**	**False Positive: 24**
**Not NAE on MK**	**False Negative: 26**	**True Negative: 38,832**

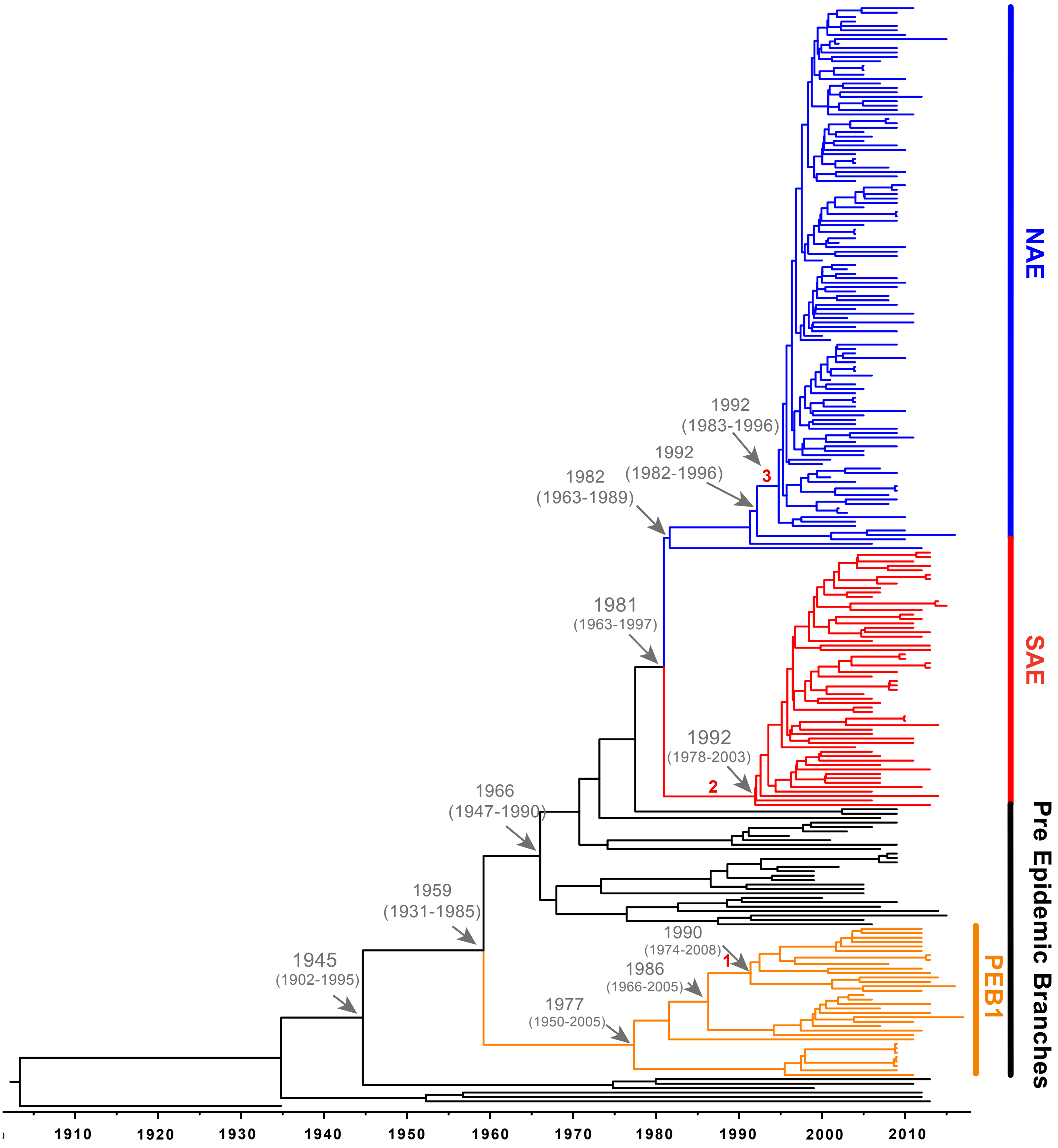

Figure S1. Bayesian maximum clade tree calculated from 305332 sampled trees using a relaxed clock. Tree generated using uncorrelated lognormal relaxed clock and a constant-size coalescent population. Four independent runs of 200 million MCMC steps with a 5,000-step thinning were completed. For each run, 10% of the first posterior samples were removed as a burn-in and the posterior estimates from 4 individual runs were combined to form a posterior sample density based on 760 million samples. Important MRCA are indicated as dates (95% HPD) and introduction of key genetic elements are labelled (1. vSaß loss, 2. COMER acquisition, 3. ACME acquisition)

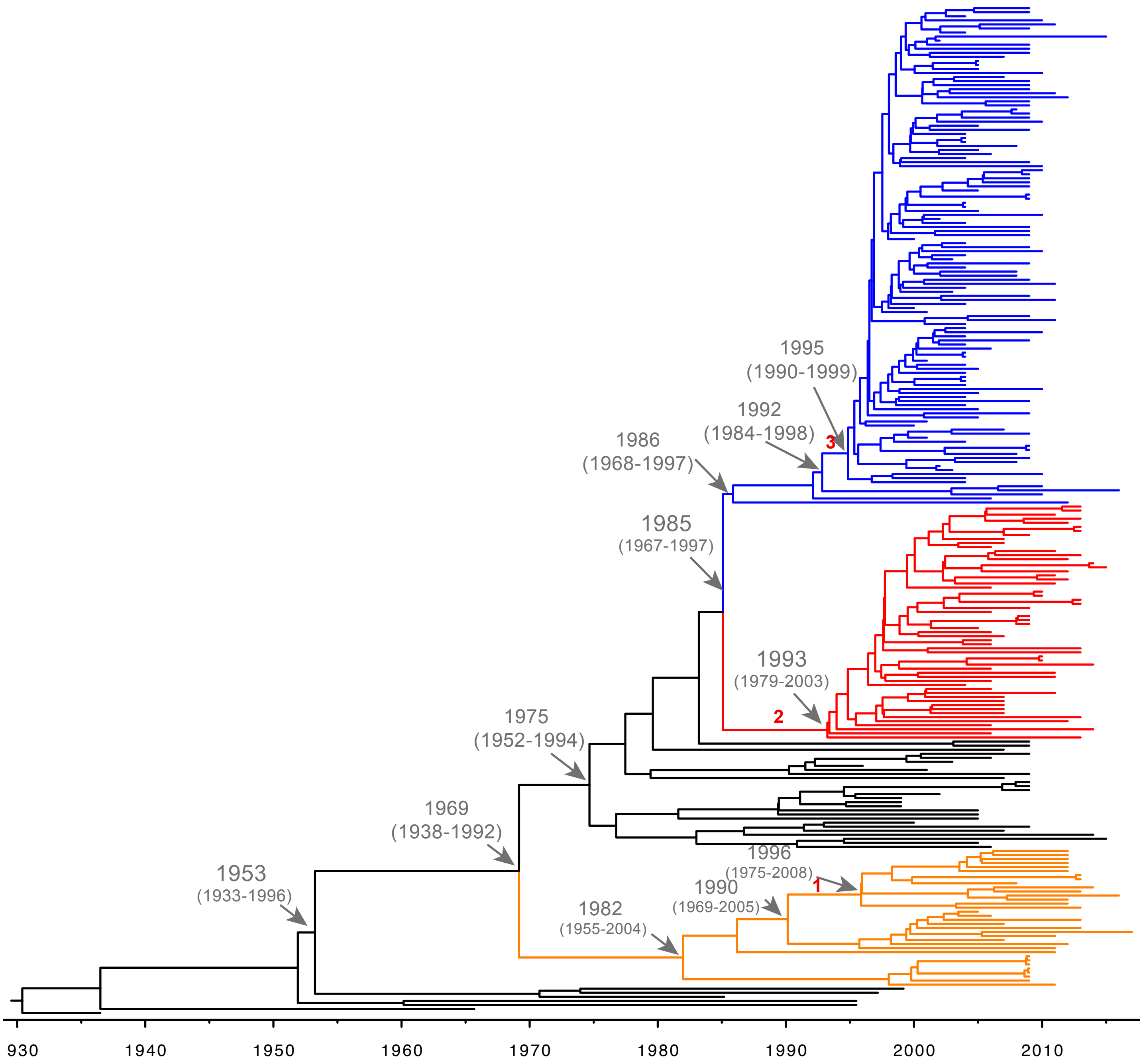

Figure S2. Bayesian maximum clade tree calculated from 148936 sampled trees using a relaxed clock. Tree generated using uncorrelated lognormal relaxed clock and a coalescent exponential population. Two independent runs of 200 million MCMC steps with a 5,000-step thinning were completed. For each run, 10% of the first posterior samples were removed as a burn-in and the posterior estimates from 2 individual runs were combined to form a posterior sample density based on 380 million samples. Important MRCA are indicated as dates (95% HPD) and introduction of key genetic elements are labelled (1. vSaß loss, 2. COMER acquisition, 3. ACME acquisition)

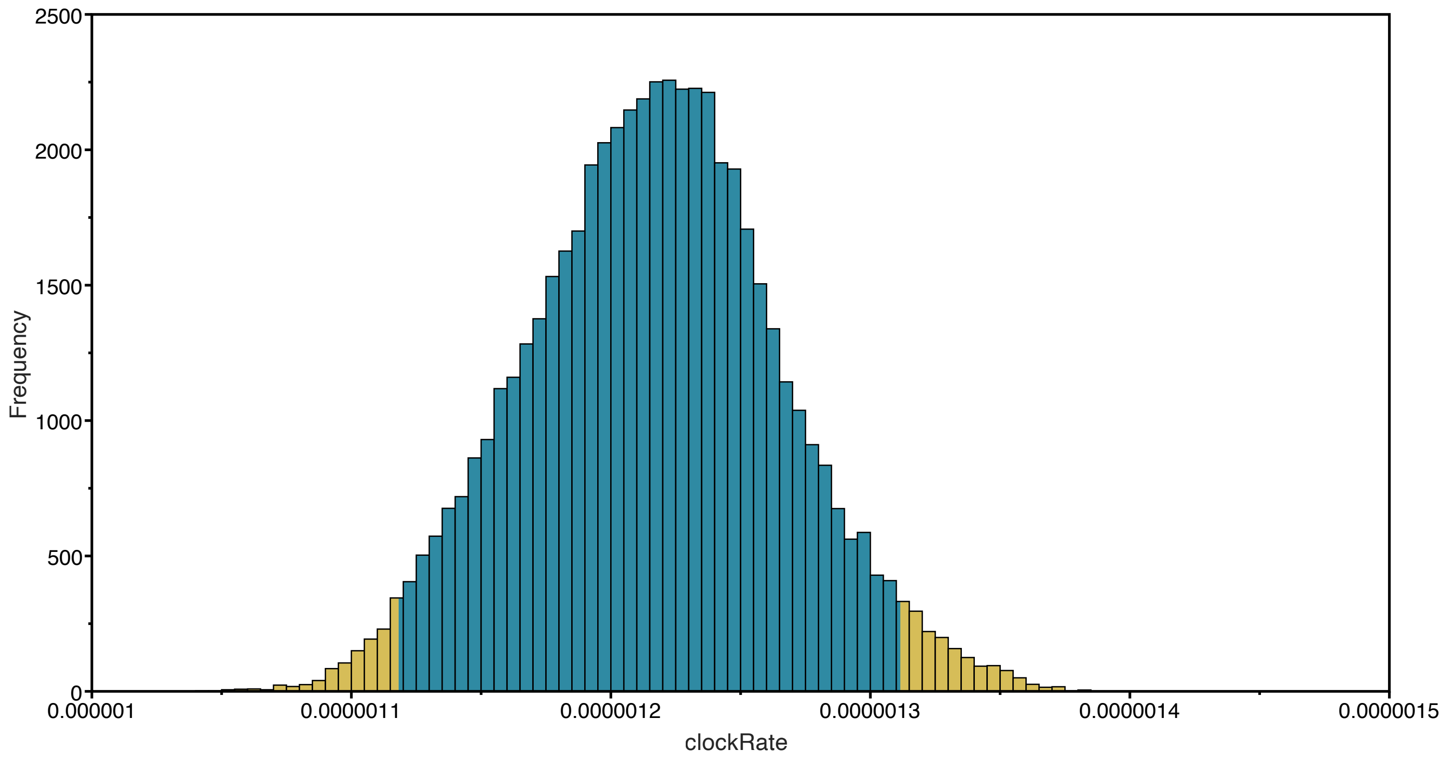

Figure S3. Mean evolutionary rate (substitutions/site/year) across the whole phylogenetic tree using a strict clock model with a random starting tree and a coalescent constant population. Mean=1.2164E-6, Median=1.217E-6, SE=4.6231E-9, SD=4.8944E-8, 95% HPD Interval=1.1184E-6, 1.3117E-6.

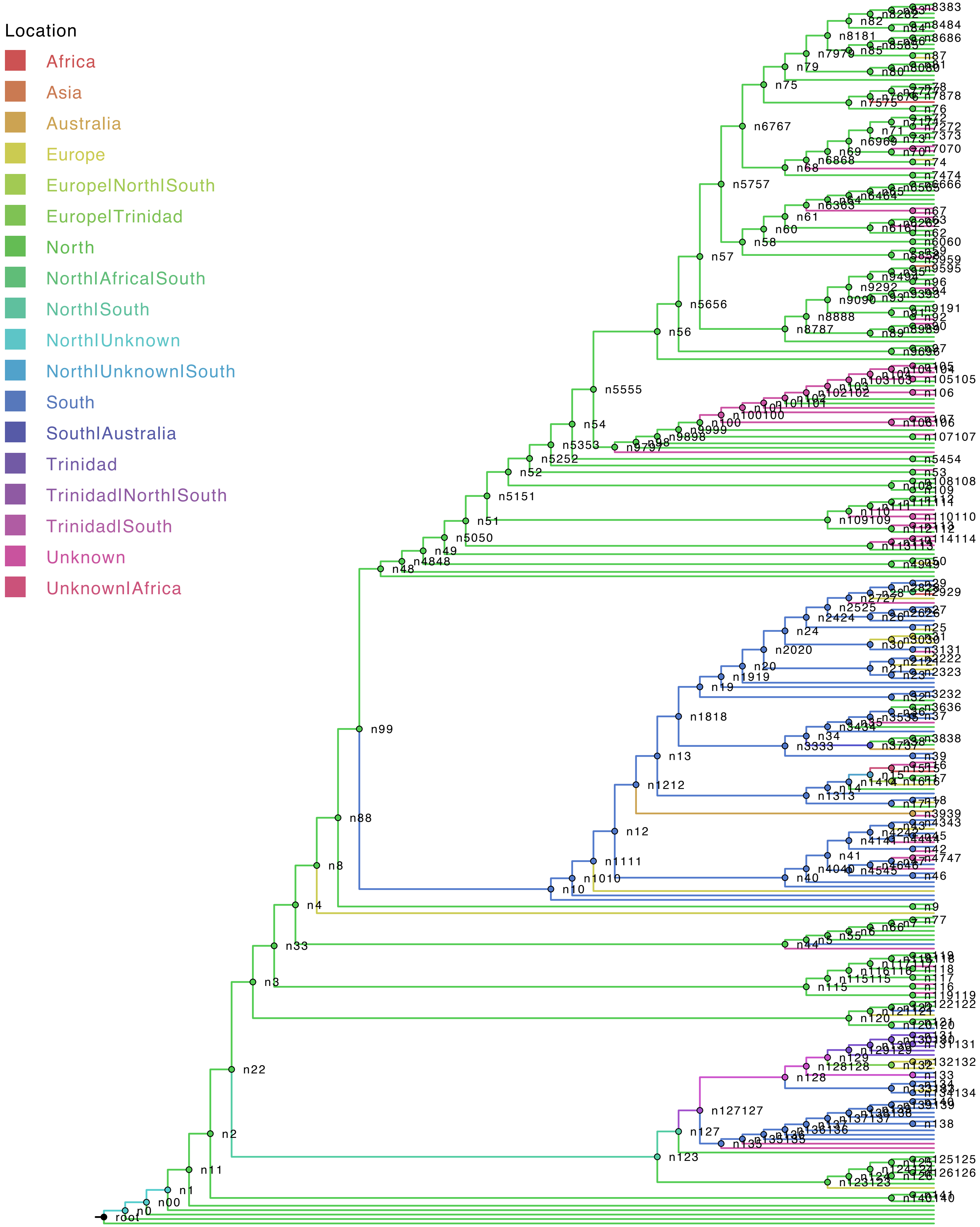

Figure S4: Ancestral state reconstruction was done as described in Fig. 3. To test for the impact of sampling bias we randomly chose 11 EB genomes from North America and reassigned their geographical location as “unknown”, resulting in an equal number of EB genomes (17 each) with North American and South American assignments. We repeated this procedure 10 times. Nodes are labeled and corresponding probabilities of location for each node in the EB are in Table S3.

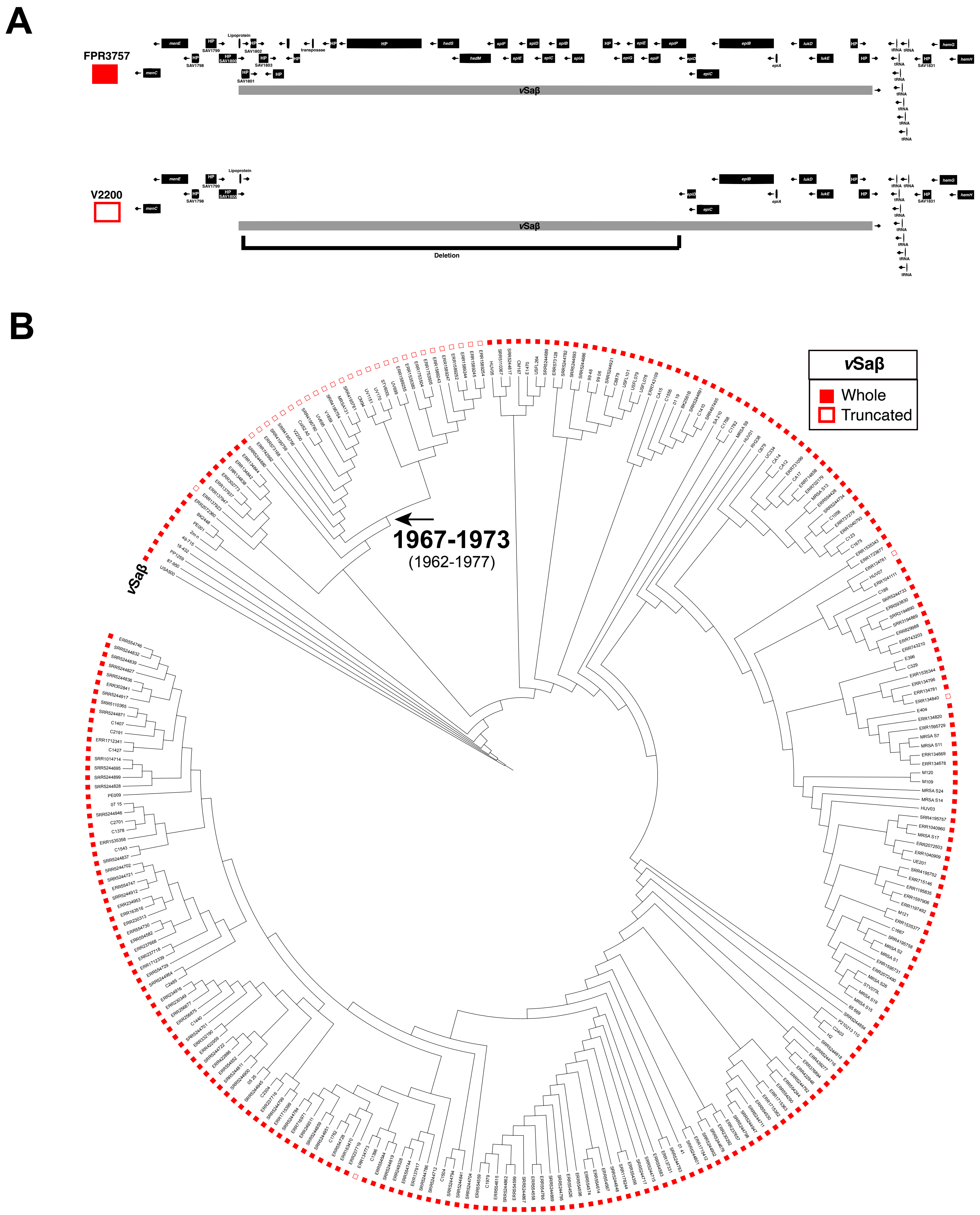

Figure S5: Majority of genomes in the PEB1 are missing part of the genomic island vSaβ, most likely through an excision event. (A) Representative alignment of whole (FPR3757) or truncated (V2200) genomic island vSaβ. Arrows show orientation of open reading frames. HP= hypothetical protein. (B) Phylogenetic analysis of 276 S. aureus strains showing the presence or absence of whole vSaβ in USA300. Estimated date of excision is indicated (95% HPD).

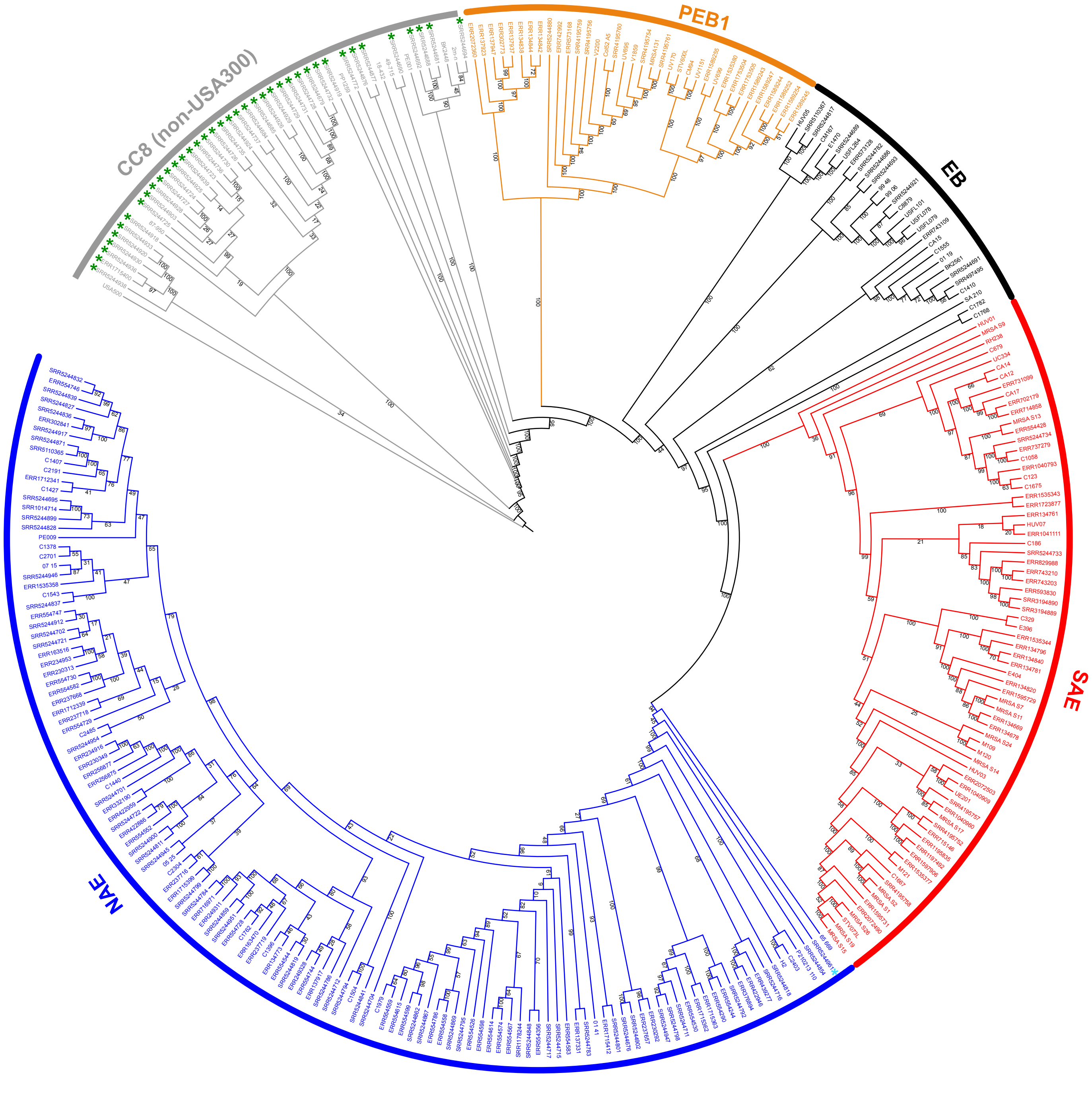

Figure S6: Phylogenetic analysis of USA300 and non-USA300 CC8 genomes: Branches and names are colored according to clade. Additional CC8 genomes in this analysis are marked with a green asterisk and the added “SRR5244961” genome is marked with cyan asterisk.

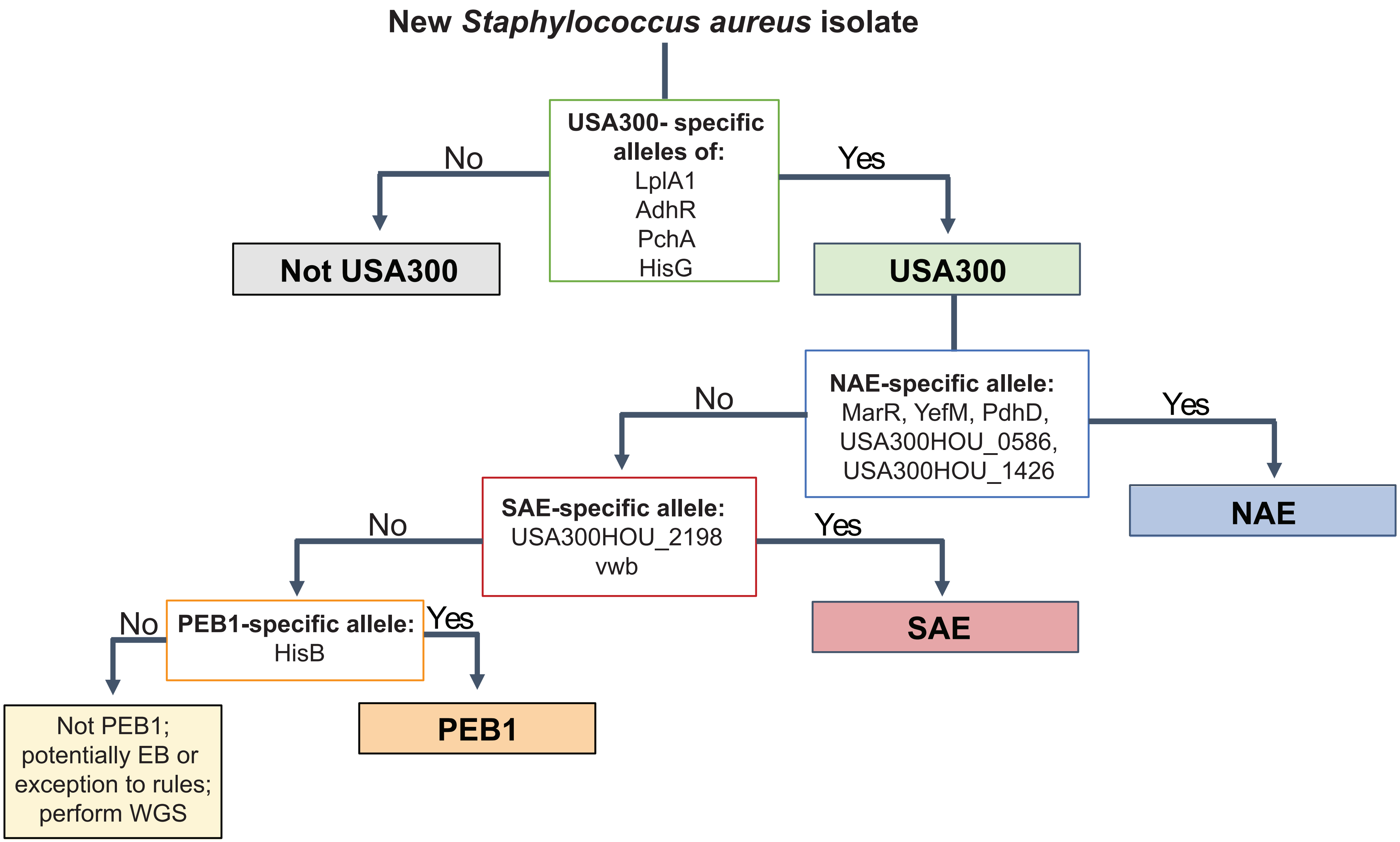

Figure S7: Suggested process for identifying a newly isolated *Staphylococcus aureus*. Alleles are specific to each clade and do not need to be used in conjunction with a prerequisite. For example, you can test for PEB1 without first determining that your isolate is not NAE. The order suggested reflects the probability that a new isolate will most likely be NAE, then SAE, and finally PEB1.

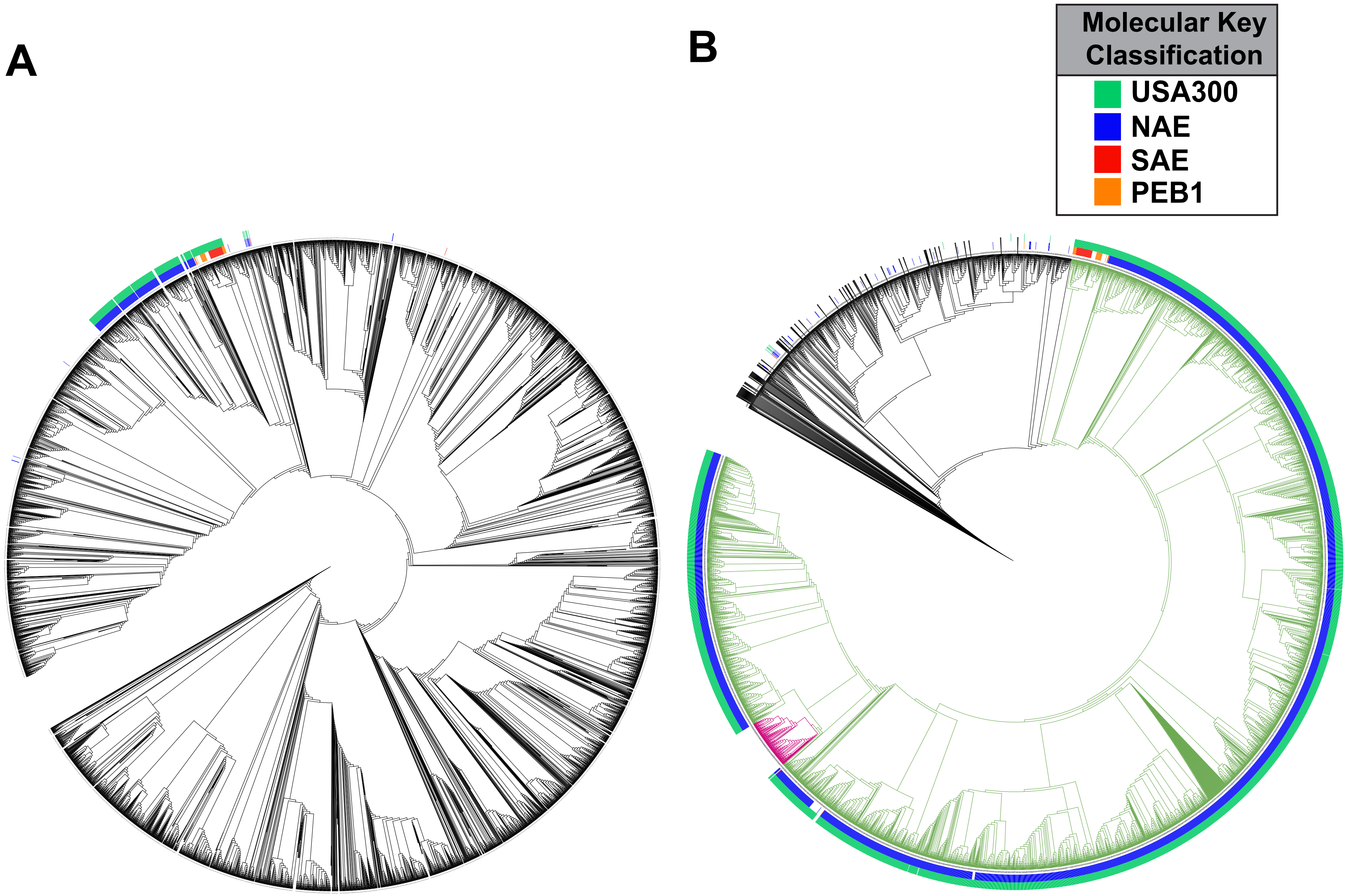

Figure S8: Neighbor joining tree of all 42949 S. aureus genomes on Staphopia. Genomes classified as USA300 using the allele-based molecular key are labeled with green (inner ring) and genomes possessing clade-specific alleles are colored according to alleles used (outer ring). A. Is the fully expanded tree. B. is the same tree with all clades besides the putative USA300 clade (green branches) collapsed. The clade in located within USA300 but not identified as USA300 using the molecular key is labeled in pink.

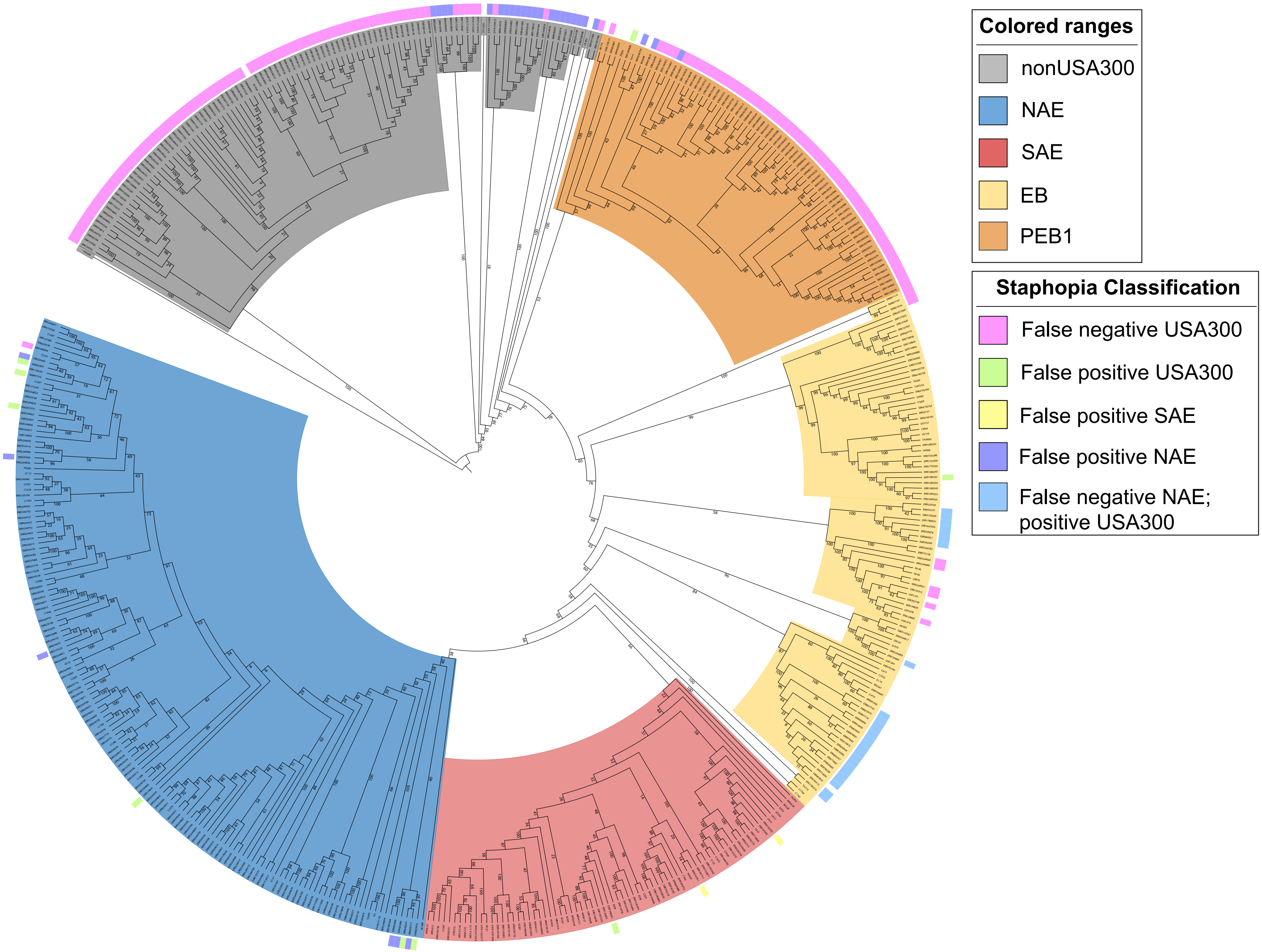

Figure S9. Maximum likelihood tree with added conflicting *S. aureus* genomes: Genomes for which the classification using the allelic molecular key were discordant with the relationships based on the NJ Staphopia tree were re-assessed using maximum likelihood in combination with 276 genomes from Fig. 1. The branches are colored blue for NAE, red for SAE, yellow for early branching clades and PEB1, and gray for non-USA300 CC8. The outer ring color indicates the results of the initial analysis based on classification using the allelic molecular key as the "experiment" and Staphopia tree as "true."
